# Clinical assessment of peripheral arterial occlusive disease and various classifications

**DOI:** 10.1007/s12055-025-02055-x

**Published:** 2025-10-25

**Authors:** Seyed Pouriya Hosseini Mehr, Reza Sarwary, Ezigbo Iyamah, Theodore Patel, Raghvinder Pal Singh Gambhir

**Affiliations:** Department of Vascular Surgery, King’s College Hospital NHS Foundation Trust, Denmark Hill, London, SE5 9RS UK

**Keywords:** Peripheral arterial occlusive disease (PAOD), Atherosclerosis, Chronic limb-threatening ischaemia (CLTI), WIfI classification, GLASS classification

## Abstract

With an ageing population and increasing prevalence of risk factors like diabetes mellitus, there is a worldwide increase in the prevalence and incidence of peripheral arterial occlusive disease (PAOD). Risk factors for PAOD include, among others, smoking, diabetes, hypertension and hyperlipidaemia. Early diagnosis and management are essential to reduce the risk of amputation and cardiovascular mortality. Keeping up with improved diagnostic and interventional capabilities, different classification systems have evolved over the years to assess the severity, guide treatment strategies and predict the outcomes.

## Introduction

Peripheral arterial occlusive disease (PAOD) is a chronic progressive condition which can lead to narrowing and eventual blockage of the peripheral arteries. It more commonly affects the lower limbs, though it can also affect the upper limbs. PAOD is typically caused by atherosclerosis or inflammatory changes in the blood vessels, reducing blood flow and limiting tissue perfusion. Other aetiologies include embolism, trauma and vasculitis.

PAOD is a global health problem with an estimated prevalence of 3–10%, increasing to 15% in persons over 70 years of age [1]. Using a definition of peripheral arterial disease as an ankle brachial index (ABI) lower than or equal to 0.90, it is estimated to affect > 250 million people worldwide, with a majority living in low-income and middle-income countries [2, 3]. Unlike the high-income countries, where the incidence increases with age, in Southeast Asia and the West Pacific region, most cases are reported in people younger than 55 years [2].

The PAOD-related amputation rate varies between 3.6 and 68.4 per 100,000 per year across the world [4]. Access to healthcare seems to play a crucial role in patient’s outcomes, with lower amputation rates in high-income countries (determined by gross domestic product (GDP) per capita) where patients may receive earlier and more effective interventions, whilst in lower-income countries, delays in diagnosis and access to treatment may contribute to higher amputation rates [5, 6].

Besides the obvious risk of amputation, patients with PAOD have a threefold increased risk to die from all causes and a sixfold increased risk to die from cardiovascular disease within a period of 10 years compared with patients without PAOD [5, 7].

## Risk factors

Risk factors for PAOD include male gender, hypertension, high cholesterol, diabetes mellitus (DM), previous and current smoking, chronic kidney disease, elevated homocysteine levels and a family history of atherosclerosis [1].

## Diabetes and PAOD

Around 8.0% of the global population is projected to be diagnosed with impaired glucose tolerance by 2030 [8]. Diabetes mellitus (DM) itself remains a major risk factor for PAOD. Patients with DM have more than two-fold increased prevalence of PAOD compared with the general population. These patients also have worse outcomes in terms of lower extremity complications such as amputations and ulcers, compared to patients with DM or PAOD alone [9, 10].

Left untreated, the overall risk of limb loss in chronic limb-threatening ischaemia (CLTI) is estimated at approximately 20–25% at 1 year, with a huge impact on quality of life and healthcare costs [2]. More than half of people with a major amputation will die in 5 years [11].

Furthermore, patients with concurrent established PAOD and DM are more likely to have major adverse cardiovascular and cerebrovascular events compared to non-DM patients. Even when successfully revascularised, there is an increased risk of graft and bypass failure in diabetic patients [12].

Worldwide, the incidence of DM-related limb amputations is increasing [13]. An editorial in *Lancet* in 2005 stated, “someone, somewhere, loses a leg because of diabetes every 30 s of every day” [14]. This is estimated to be every 20 s now. Amputations have a huge social and economic consequence due to decrease in quality of life and life expectancy, and increase in the healthcare costs.

Eighty percent of amputations are preceded by ulcers; therefore, the importance of diligent clinical assessment through history taking and comprehensive clinical examination before ordering appropriate investigations is emphasised.

## Clinical assessment

**Fig. 1:**
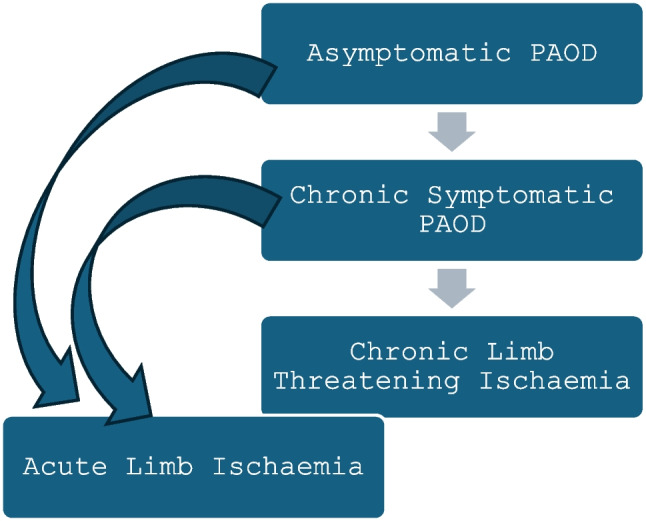
PAOD: clinical subsets

Assessment through detailed clinical history and physical examination is central to management of PAOD. There are four clinical subsets of presentations, each with a varying degree of urgency of care [15] (Fig. 1).

## History

A comprehensive medical and social history including the cardiovascular risk factors, drug history, family history of vascular diseases, dietary habits, physical activity levels and history of previous vascular and endovascular revascularisation procedures and amputations is essential before making management decisions. Importantly, smoking history should include both current and past smoking pack years. Assessment of frailty, functional status and health-related quality of life (HRQL) is essential to make patient-centred treatment plans.

Presentation of PAOD may range from asymptomatic disease to intermittent claudication in chronic symptomatic PAOD and, in more severe forms, CLTI with rest pain or tissue loss. Leg cramps, wounds that do not heal, tingling, numbness and coldness or discoloration of the toes may be the initial presentation.

Claudication (derived from the Latin word meaning “to limp”) is the hallmark of chronic symptomatic PAOD and can significantly affect the quality of life. It is a reproducible ischaemic muscle pain (ache/cramp) which is primarily triggered by physical activity when the blood flow to the muscles is insufficient during exertion and is relieved by short rest. The pain occurs one segment below the level of stenosis or occlusion and typically affects the calf or thighs or buttocks. A patient typically complains of the calf muscle “seizing up” after walking a certain distance (approximately 100–200 yards) every time. This distance is the claudication distance and with progression of disease, the claudication distance keeps decreasing and the time to recover keeps increasing and the patient is able to walk even less after rest.

As the disease progresses, the patient may develop short-distance claudication (~ 25 yards) or can develop ischaemic rest pain, which usually affects the forefoot and is frequently worse at night on leg elevation. The pain wakes up the patient at night and is relieved by lowering the legs. It can become so debilitating that the patient has to sleep in a chair. Rest pain is often a precursor for the development of ulcers and gangrene. Ischaemic ulceration and gangrene are typically distal in location affecting the toes and forefoot.

In patients with diabetic neuropathy, due to altered biomechanics and foot deformity, pressure-bearing areas of the foot are affected. It is important to remember that diabetic patients with profound neuropathy may not complain of ischaemic rest pain; hence, they may present with greater necrosis at presentation.

## Physical examination

Patients with chronic ischaemia often have thin, shiny, dry skin due to loss of subcutaneous fat along with loss of hair and dystrophic toenails, and may go on to develop muscle atrophy.

Presence of the so-called sunset foot is a hallmark of CLTI; however, examination with leg dependent may give a false impression of good capillary refill and adequate perfusion. Buerger’s sign—pallor of the foot on elevation and dependent rubor—should be an essential part of examination. Buerger’s angle is a good predictor of the severity of the disease.

Additional things to look for during limb inspection include previous scars indicating previous vascular procedures/saphenous vein harvesting. Also look for trophic skin lesions, micro emboli (trash foot) and livedo reticularis.

On palpation, the part feels cool, capillary refill time and venous refilling times are increased and there is venous guttering on leg elevation.

Palpation of bilateral lower limb pulses—femoral, popliteal, dorsalis pedis and posterior tibial—is essential to assess peripheral circulation. A palpable pulse typically indicates adequate perfusion. Atrial fibrillation (AF) can also be picked up during pulse assessment. Audible bruit in the femoral artery, or over the abdominal aorta, signifies stenotic disease.

Neurosensory examination using monofilaments is a must in patients with DM and may reveal “glove and stocking” neuropathy. Evaluation with a tuning fork may reveal loss of vibration sense, which is an early sign. In diabetic patients with a foot sinus/ulcer, a probe-to-bone test should be performed to assess depth and the probability of underlying osteomyelitis.

A handheld Doppler device is a valuable bedside tool for the quick evaluation of peripheral circulation if the peripheral pulses are difficult to appreciate. Ankle brachial index (ABI) measurement should be considered an extension of the physical examination. It provides a reasonable measure of perfusion and is often used in the community to guide referrals to vascular surgery. It is important to note that calcification in DM may result in false high readings, which can be partially addressed by measuring post-exercise ABI [16].

Patients with any abnormal findings during physical assessment, or those with high risk of PAOD, should undergo further diagnostic imaging. Non-invasive investigations such as Doppler ultrasound (DUS) are often the first-line imaging investigation followed by cross-sectional imaging using computed tomography angiography (CTA) or magnetic resonance angiography (MRA) to detect the degree and level of arterial disease and existing collateral circulation.

### Chronic limb-threatening ischaemia (CLTI)

It is a clinical syndrome defined by the presence of tissue loss (ulcer/gangrene) or ischaemic rest pain in the presence of objectively proven arterial disease (absolute ankle pressure < 50 mmHg, absolute toe pressure < 30 mmHg) [1].

### Acute limb ischaemia (ALI)

It is defined as a sudden decrease in limb perfusion causing a potential threat to limb viability. Characteristic physical findings of ALI include the 5Ps—acute onset of pain, pallor, pulselessness, paraesthesia/paresis and paralysis in the affected limb. The part is perishingly cold. Mottling is a late sign.

Rutherford’s ALI classification divides an extremity into viable, threatened or irreversible categories (Table 1).

**Table 1:** Rutherford acute limb ischaemia classification

	Viable Class I	Threatened Class IIa	Threatened Class IIb	Irreversible Class III
**Clinical description**	Not immediately threatened	Salvageable	Salvageable if treated promptly	Non-salvageable
Capillary refill	Intact	Slow	Slow	None, marbling
Sensation	Normal	Affected	Affected	Lost
Motor function	Normal	Normal	Decreased	Lost, paralysed
Arterial Doppler signals	Audible Ankle pressure > 30 mmHg	Inaudible	Inaudible	Inaudible
Venous Doppler signals	Audible	Audible	Audible	Inaudible

## Comparative analysis and clinical utility of various classification systems

**Fig. 2:**
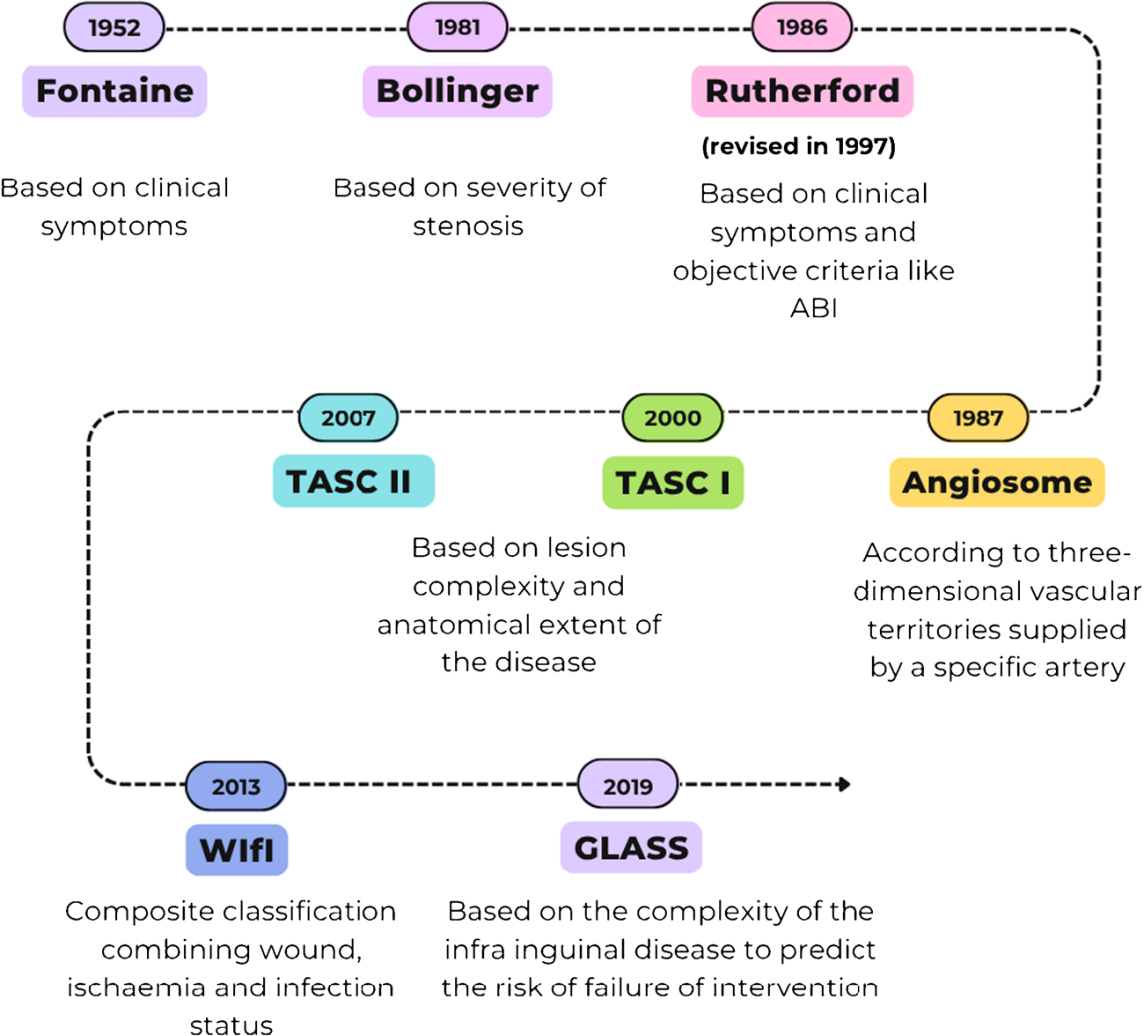
Chronology of peripheral arterial disease classification systems. Abbreviations: TASC, Trans-Atlantic Inter-Society Consensus for the management of peripheral arterial disease classification; WIfI, wound, ischaemia and foot infection; GLASS, gobal limb anatomic staging system

Accurately staging the severity of arterial disease is fundamental to clinical decision-making in PAOD and predicting patient outcomes. A number of classification systems have evolved over the years based on symptoms or location and severity of individual arterial lesions [17–22] (Fig. 2). Each classification system has its strengths and limitations. Lesion or segment-based anatomical classification systems are useful when comparing endovascular treatment outcomes. Anatomic classification systems have usually been based on catheter-directed angiography.

## Rutherford and Fontaine peripheral arterial disease classifications

Fontaine gave the initial classification of PAOD in the 1950 s based on clinical symptoms alone [12]. As the availability of non-invasive haemodynamic testing increased, Rutherford published his classification in 1986, and it included objective haemodynamic criteria like ankle and toe pressures, which gave it more accuracy and reproducibility (Table 2) [18].

**Table 2:** Fontaine and Rutherford classifications

Rutherford classification	Clinical description	Objective criteria	Equivalent Fontaine stages
Category 0	Asymptomatic	Normal ABI	Fontaine stage I
Category 1	Mild claudication	ABI < 0.90	Fontaine stage IIa Claudication distance > 200 m
Category 2	Moderate claudication	ABI < 0.70	Fontaine stage IIb Fontaine stage IIb Claudication distance < 200 m
Category 3	Severe claudication	ABI < 0.40	
Category 4	Ischaemic rest pain	ABI < 0.40	Fontaine stage III
Category 5	Minor tissue loss	ABI < 0.40	Fontaine stage IV
Category 6	Major tissue loss	ABI < 0.40	

## TASC

**Fig. 3:**
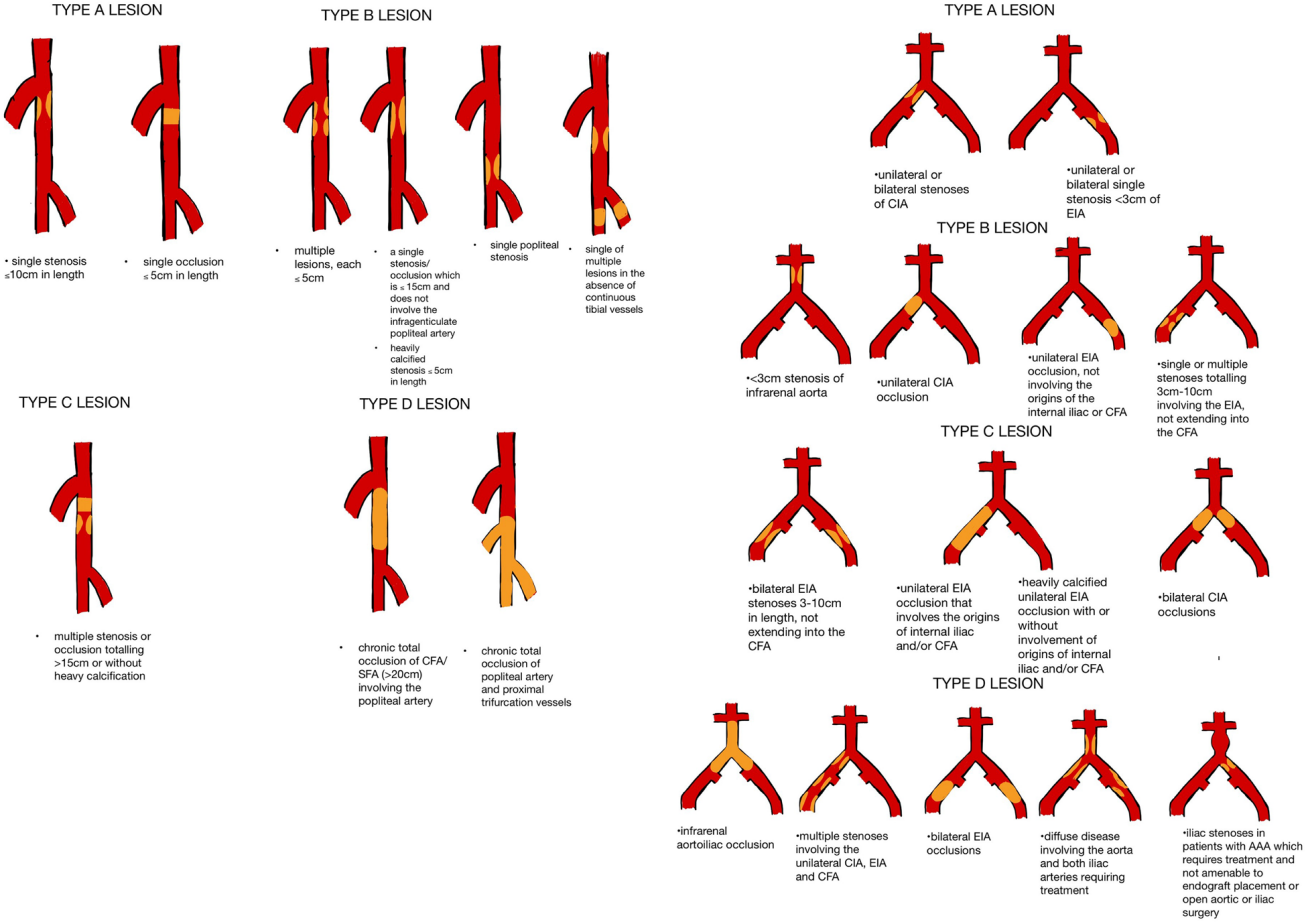
The Trans-Atlantic Inter-Society Consensus for the management of peripheral arterial disease classification femoropopliteal and aorto-iliac systems (TASC II)

The first Trans-Atlantic Inter-Society Consensus for the management of peripheral arterial disease classification (TASC) system was the result of international collaboration between 14 different societies [16]. The first edition of the TASC guidelines was released in 2000, and an updated version, which incorporated new guidelines and streamlined the information for diagnosis and management, was released in 2007 (Fig. 3) [1]. At the time, it was thought that TASC A and B lesions were more suitable for endovascular treatment and TASC D for open surgical interventions.

## The society for vascular surgery (SVS) WIfI classification system

**Fig. 4:**
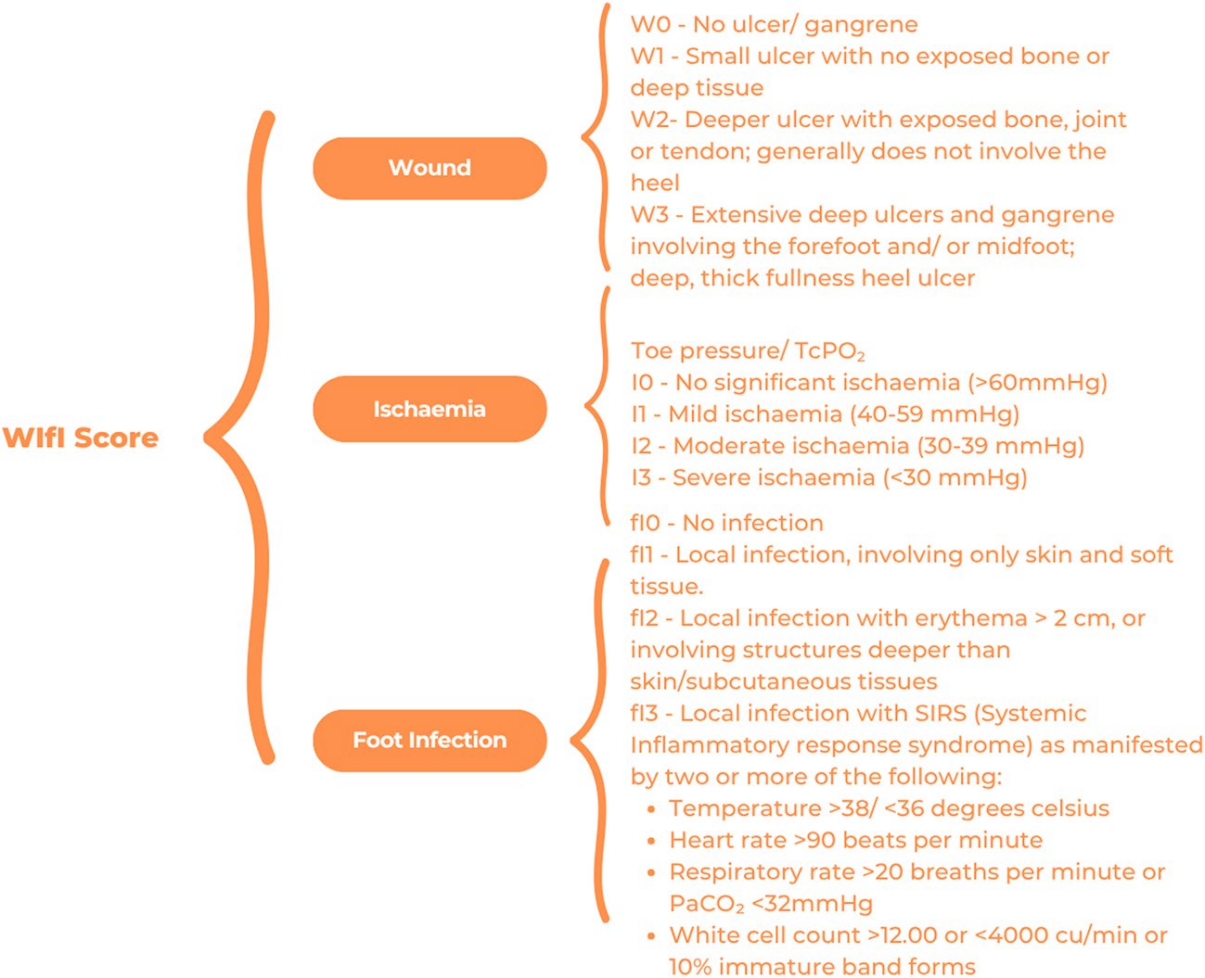
Grading in the wound, ischaemia and foot infection (WIfI) system

The wound, ischaemia and foot infection (WIfI) classification was developed in 2013 and provides an objective classification for wound healing and limb amputation based on three independent risk factors—wound extent, degree of ischaemia and extent of foot infection [20]. The WIfI system categorises patients in a system similar to cancer TNM staging. A separate grade is given to the wound (the presence and depth of ulcer), ischaemia (based on ABI, toe pressure, or transcutaneous oximetry (TcPO2)), and infection (local to systemic) [21] (Fig. 4). The three grades are combined to give a risk of amputation, likelihood of wound healing and estimated benefit of revascularisation.

## Global Limb Anatomic Staging System (GLASS) classification

The Global Limb Anatomic Staging System (GLASS) was published in 2019 by the Global Vascular Guidelines to facilitate the development of appropriate evidence-based revascularisation (EBR) strategies in patients with CLTI. It aims to get away from anatomic/lesion/segment-based classifications.

Establishment of direct pulsatile in-line flow is essential for effective revascularisation and wound healing in CLTI. Based on angiographic images, a target arterial path (TAP) is defined by the vascular specialist to provide straight line flow to the foot. Estimated limb-based patency (LBP) is then defined as maintenance of in-line flow throughout the TAP, from groin to ankle. The aorto-iliac inflow disease (Table 3) is presumed to be corrected, and femoropopliteal (FP) (Table 4) and infra-popliteal (IP) (Table 5) arterial segments are graded on a scale of 0 to 4 and then using a matrix combined into three overall GLASS (I–III) stages for the limb. The infra-malleolar (IM)/pedal disease grades (Table 6) are not used in the matrix (Table 7) [22].

**Table 3:** Aorto-iliac disease staging in GLASS

	Common femoral artery disease	Infra renal aortic disease	Common iliac artery (CIA)	External iliac artery (EIA)	Combinations
1A 1B	Not significant Significant > 50%	Stenosis	Stenosis/CTO	Stenosis/CTO	Any except combined CTO of CIA and EIA
2A 2B	Not significant Significant > 50%	CTO	CTO or severe disease with < 6 mm calibre	CTO or severe disease with < 6 mm calibre	Concomitant

*CTO*, chronic total occlusion

**Table 4:** Femoro-popliteal disease staging in GLASS

Femoral popliteal grade (FP)	Total SFA length	SFA descriptor	Popliteal artery disease
FP0		< 50% disease	Nil
FP1	< 10 cm (1/3rd)	< 5 cm CTO No flush occlusion	Mild/no disease
FP2	10–20 cm (1/3–2/3rd)	< 10 cm occlusion	< 2 cm focal stenosis Trifurcation spared
FP3	> 20 cm	Flush CTO < 20 Non-flush CTO 10–20 cm	2–5 cm stenosis Trifurcation spared
FP4	> 20 cm		> 5 cm stenosis or trifurcation involved Any CTO

*CTO*, chronic total occlusion

**Table 5:** Infra-popliteal disease staging in GLASS

Infra-popliteal grade (FP)	Tibial artery stenosis/disease	Descriptor
IP0	Mild/none	
IP1	< 3 cm focal stenosis	No flush occlusion
IP2	1/3rd length	< 3 cm CTO Not flush TP trunk spared
IP3	< 2/3rd length	CTO < 1/3rd Flush lesion TP trunk spared
IP4	> 2/3rd length	CTO > 1/3rd CTO of TP trunk if AT is not TAP

*CTO*, chronic total occlusion

**Table 6:** Pedal/infra-malleolar (IM) disease staging in GLASS

Pedal/infra-malleolar grade (P)	Target artery crosses ankle into foot	Pedal arch
P0	Yes	Intact
P1	Yes	Absent/severely diseased
P2	No

**Table 7:** Analysis based on GLASS stages

Stage	Technical complexity	Pattern of disease FP grade and IP grade	Estimated technical failure	Estimated 1-year limb-based patency
Stage I	Low	FP0;IP1/2 FP1;IP1/2 FP2;IP1	< 10%	> 70%
Stage II	Intermediate	FP3;IP0/1/2 FP2;IP1/2/3 FP1;IP2/3 FP0;IP3	< 20%	50–70%
Stage III	High	FP4; IP 0–4 FP0-4;IP4, FP3, IP3	> 20%	< 50%

GLASS stages for the limb thus reflect the complexity of the infrainguinal disease and predict the risk of immediate technical failure (ITF) of intervention and chances of LBP in each stage.

**Fig. 5:**
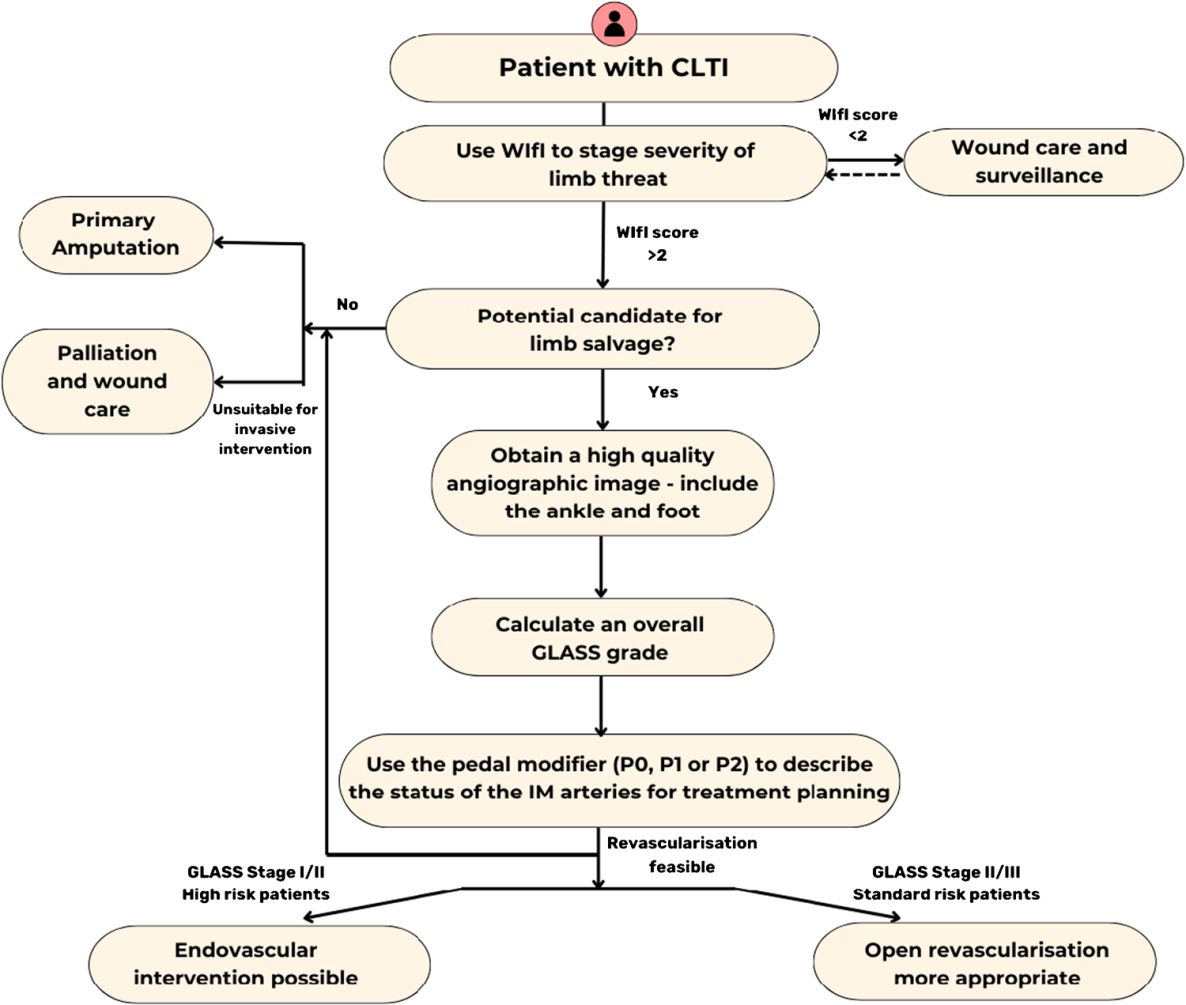
Framework of clinical decision-making in chronic limb-threatening ischaemia (CLTI) with integrated wound, ischaemia and foot infection (WIfI) and Global Limb Anatomic Staging System (GLASS) approach

The relevance of these GLASS anatomic stages in different clinical scenarios is integrated within the PLAN (Patient risk estimation, limb staging, ANatomic pattern) framework for decision-making (Fig. 5).

With universal use of WIfI classification and GLASS, there is hope that we have better tools to stratify the severity of limb-threatening PAOD and incorporate patient risks into management decisions as well as guide future research.

**Fig. 6:**
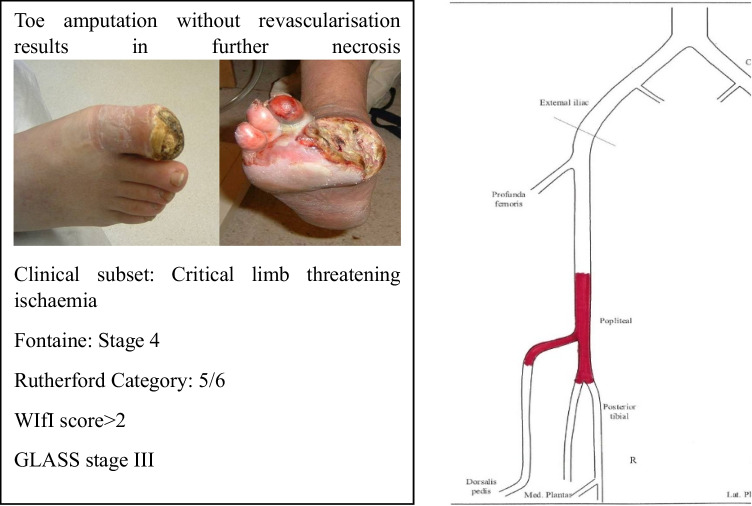
Integration of clinical assessment and classification systems at the patient level

An appropriate example of integration of clinical assessment and classification systems is highlighted in Fig. 6.

## Conclusion

Integrating clinical assessment with diagnostic imaging is essential to allow patient-tailored treatment strategy, rather than lesion-centric treatment. Early diagnosis can allow lifestyle modifications and pharmacological therapy to reduce the risk of cardiovascular complications. Current classification systems allow objective comparison of various modalities of revascularisation strategies and help improve patient outcomes.

## Data Availability

No patient data was used/stored.
